# Model of goal directed behavior for limiting Latino preschoolers’ television viewing: validity and reliability

**DOI:** 10.1186/s12889-020-8268-x

**Published:** 2020-02-05

**Authors:** Marissa Ogren, Tom Baranowski, Sarah J. Lowry, Jason A. Mendoza

**Affiliations:** 1 Department of Psychology, University of California, Los Angeles, 1285 Franz Hall, Box 951563, Los Angeles, CA 90095-1563 USA; 2 USDA/ARS Children’s Nutrition Research Center, Baylor College of Medicine, Houston, TX USA; 3 Center for Child Health, Behavior and Development, Seattle Children’s Research Institute, Seattle, WA USA; 4 Department of Pediatrics and Nutrition Sciences Program, University of Washington, Seattle, WA USA; 5 Fred Hutchinson Cancer Research Center, Seattle, WA USA

**Keywords:** Model of goal directed behavior, TV viewing, Parenting, Child health, Health behavior, Theory of planned behavior, Preschoolers

## Abstract

**Background:**

Accurately measuring parents’ attitudes and beliefs regarding limiting their children’s TV viewing is important to inform the design and evaluation of effective interventions. This manuscript assesses the internal consistency reliability, test-retest reliability, convergent validity, and construct validity of the Model of Goal Directed Behavior (MGDB) scales among parents of Latino preschoolers to characterize Latino parents’ attitudes and beliefs toward limiting their preschoolers’ TV viewing.

**Method:**

Participants included parents of Latino preschoolers in the United States, 3–5 years old (*n* = 186). Parents completed a socio-demographic survey and the 105-item MGDB questionnaire (Attitudes, Perceived Positive/Negative Behavioral Control, Subjective Norms, Positive and Negative Anticipated Emotions, Habits, Self-Efficacy, Desires, and Intentions surrounding their child’s TV viewing) which was used to measure internal consistency reliability and construct validity. A subsample of participants completed the questionnaire twice to measure test-retest reliability. Further, parents completed a 7-day TV viewing diary for their preschooler, and a TV parenting practices questionnaire as measures of convergent validity.

**Results:**

Internal consistency reliability was generally acceptable for the MGDB scales (Cronbach’s alphas> 0.7), except for the Desires scale, which was revealed to have two factors and the Attitudes and Perceived Behavioral Control scales. Test-retest reliability over 2 months had negligible to moderate correlations (*r*’s = 0.28 to 0.61). Two structural equation models were conducted. One yielded acceptable model fit (*x*^2^ (97) = 113.65, *p* = .119) and the other had questionable model fit (*x*^2^ (97) = 125.39; *p* = .028). Testing convergent validity, only two MGDB scales (Habits and Self-Efficacy) were positively correlated with the TV parenting practices questionnaire (*r*’s = 0.33 to 0.51), and none were meaningfully correlated with preschoolers’ mean daily TV viewing.

**Conclusions:**

Initial reliability and validity for some of the MGDB scales appear acceptable among parents of Latino preschoolers. Refinement of the instrument and testing among larger samples is necessary to fully evaluate psychometric properties. This instrument may be useful for characterizing Latino parents’ attitudes and beliefs toward limiting their preschoolers’ TV viewing and informing future TV reduction interventions.

**Trial registration:**

Clinical Trials NCT01216306 Registered October 6, 2010.

## Background

Childhood obesity in the US is a major health concern [1], especially among Latinos [2, 3]. Considering the long-term health risks [4], it is important to identify culturally specific obesity prevention interventions for this underrepresented group [5]. TV viewing early in life may be one contributing factor worth targeting [6–11], particularly given increased rates of TV viewing among Latino children [12]. Although it is unclear exactly why Latino children watch more TV, i.e., whether related to cultural, language, or SES influences, increasing our understanding of why Latino children engage in more TV viewing is needed. Because parents exert a strong influence on health behaviors [13], and specifically TV viewing [10, 14] of their preschool children, and over one-third of US children watch more TV than is recommended [15–17], it is important to better understand how parents influence their children’s TV viewing. In particular, understanding why some parents attempt to limit their children’s TV viewing, while others do not or are unsuccessful in this endeavor should inform effective TV reduction interventions [18, 19].

To fill these gaps, we assessed a potentially crucial social and behavioral determinant of children’s health: Latino parents’ attitudes and beliefs associated with limiting their preschool child’s TV viewing. We applied the Model of Goal Directed Behavior (MGDB) to characterize parents’ attitudes and beliefs. MGDB is a conceptual model which aims to predict which individuals are more likely to engage in health behaviors, and to better understand why they may do so. MGDB is an expansion of the Theory of Planned Behavior which adds explanatory social cognitive type variables such as anticipated emotions, desires, and past behavior [20–24] in order to predict intentions which in turn predict health behavior. With these variables, MGDB better predicted intentions to be physically active than the Theory of Planned Behavior alone [25]. The MGDB has been previously validated in other populations for different health behaviors including vegetable consumption and physical activity [21, 25], but there has been no psychometric evaluation of MGDB scales related to limiting children’s TV viewing.

To fill this gap, the present report tests the psychometrics of the MGDB in order to better understand the efficacy of such scales for measuring parental attitudes and beliefs toward their child’s TV viewing. The MGDB items for limiting preschool children’s TV viewing were adapted from the previously validated Model of Goal Directed Vegetable Parenting Practices questionnaire [21, 22]. The adaptation process included qualitative interviews with parents of Latino preschool children [26]. Themes from these qualitative interviews were used by an expert panel to modify or create relevant and culturally appropriate MGDB questions related to limiting their preschoolers’ TV viewing among a low-income Latino population [27]. Thus, it is important to assess psychometric properties of this adapted MGDB. To do so, we examined the reliability, convergent and construct validity of MGDB scales to characterize Latino parents’ attitudes and beliefs toward limiting their preschoolers’ TV viewing. Such information is crucial for gauging the utility and applicability of the MGDB as a tool for understanding parental TV-viewing attitudes and beliefs, as well as potentially creating targeted interventions for parents. To our knowledge, this is the first manuscript to use MGDB scales specifically with Latino families, as well as test the relationship of MGDB to the TV parenting practices scales.

## Method

### Participants

The present study was nested within a TV viewing reduction pilot cluster randomized controlled trial [26] in which 186 parents of Latino preschoolers were recruited from a convenience sample of six Head Start centers in the Houston-metro area. Head Start centers provide early childhood education for low-income children. To take part in the study, Head Start centers had to have at least one classroom comprised of 75% or more Latino students, which was determined based on ethnicity composition provided by the Head Start centers. All six centers that were approached enrolled in the study, and each had two classrooms randomized for separate, independent waves of the trial. Centers, rather than classrooms, were randomized to reduce the likelihood of contamination. Of the total 211 children eligible for the study, 186 (88%) enrolled in the study.

All participating preschoolers in the trial were between 3 and 5 years of age, of Latino or Hispanic ethnicity per parent report, and were attending a participating Head Start Center. To be included in the present MGDB validation study, parents of preschoolers needed to have completed the MGDB and related questionnaires as described below, resulting in 172 parent participants in the final sample. Previous research comparing objective and subjective measures of physical activity intensity and duration reported that samples of 50–99 are needed to provide stable estimates [28], suggesting that our sample size may be sufficient, although this sample size may not apply to the present study’s behavioral constructs which are distinct from physical activity. 52% of participants were fathers, and the average parental age was 31.3 years (*SD =* 6.9). 64% of parents reported an annual household income of $20,000 or less, and 65% of parents reported not having a high school diploma or equivalent. Further, analyses revealed no demographic differences between participants included in the MGDB validation study and those excluded due to a lack of MGDB completion (*n* = 14; Table 1). Participants received $80 for their enrollment in the trial, and provided written informed consent, which was offered in both English and Spanish.

**Table 1:** Table depicting demographic information of participants in the initial randomized controlled trial, separated into those included in and excluded from the present MGDB validation analyses

	Excluded from validation study	Included in validation study	*p*-value
	*n* = 14	*n* = 172	
	N (%)	N (%)	
Parent’s sex			
Male	0 (0.0%)	89 (51.7%)	0.15
Female	2 (14.3%)	83 (48.3%)	
(missing)	12 (85.7%)	0 (0.0%)	
Parent education			
8th grade or less	1 (7.1%)	75 (43.6%)	0.54
Some high school	0 (0.0%)	37 (21.5%)	
High school graduate or higher	0 (0.0%)	57 (33.1%)	
(missing)	13 (92.9%)	3 (1.7%)	
Child language			
Only Spanish	1 (7.1%)	53 (30.8%)	0.92
Spanish better than English	1 (7.1%)	64 (37.2%)	
Both equally	0 (0.0%)	26 (15.1%)	
English better than Spanish	0 (0.0%)	26 (15.1%)	
Only English	0 (0.0%)	3 (1.7%)	
(missing)	12 (85.7%)	0 (0.0%)	
Parent language			
Only Spanish	2 (14.3%)	79 (45.9%)	0.80
Spanish better than English	0 (0.0%)	45 (26.2%)	
Both equally	0 (0.0%)	30 (17.4%)	
English better than Spanish	0 (0.0%)	15 (8.7%)	
Only English	0 (0.0%)	2 (1.2%)	
Other	0 (0.0%)	1 (0.6%)	
(missing)	12 (85.7%)	0 (0.0%)	

Missing values indicate questions to which parents chose not to respond

### Measures

All questionnaires were provided to participants in both English and Spanish, and were completed in their preferred language. Time 1 data were collected in waves from fall of 2010 to fall of 2012.

#### Model of Goal-Directed Behavior (MGDB)

MGDB questionnaire items (Additional file 1) queried parental attitudes and beliefs related to limiting the TV viewing of their preschool-aged child. This questionnaire was completed at Time 1, prior to group randomization, as well as at Time 2, immediately after the intervention period, i.e., approximately two months after Time 1. For the present study, we analyzed only Time 1, baseline data (utilizing data from all participants), except for test-retest reliability in which Time 1 and Time 2 data were used for participants in the control condition only (*n* = 79).

The 105-item MGDB questionnaire consisted of eight scales, each intended to address the broad question in parentheses: Attitudes (15 items: What outcomes would you expect if your child watched less TV?), Perceived Positive/Negative Behavioral Control (17 items: How easy would it be to get your child to watch less TV?), Subjective Norms (9 items: How do important people in your child’s life feel about your child watching TV?), Positive and Negative Anticipated Emotions (PNAE; 29 items: How would you feel if you asked your child to watch less TV and they did/didn’t comply?), Habits (9 items: How often do you engage in particular TV-related behaviors without thinking about it?), Self-Efficacy (14 items: How confident are you that you can limit your child’s TV viewing?), Desires (7 items: Do you want to limit your child’s TV viewing?), and Intentions (10 items: Do you plan to limit your child’s TV viewing in the next month?), as proposed by Perugini and Bagozzi [23], and the additional constructs of Habits and Self-Efficacy as proposed by Hingle, Baranowski, and colleagues [21, 22]. Four of these scales (attitudes, subjective norms, perceived behavioral control, and intentions) were based on the Theory of Planned Behavior, and the other four scales (positive and negative anticipated emotions, habits, self-efficacy, and desires) were non-Theory of Planned Behavior expansions included in MGDB.

Parents were given three categorical responses, of which they were instructed to select the one that best described themselves and their child. In some cases, they were asked to answer how easy a particular statement would be (0 = difficult, 1 = neither easy nor difficult, 2 = easy). For other items, they were asked how much they agreed or disagreed with given statements (0 = disagree, 1 = neither agree nor disagree, 2 = agree), how often they did the listed activities (0 = never, 1 = sometimes, 2 = always), or how sure they were that they were able to perform particular tasks (0 = not sure, 1 = somewhat sure, 2 = sure).

Within each of the eight scales, a higher score indicated greater support for limiting their child’s TV viewing, with possible values ranging from 0 to 2. Some individual questions were reverse coded so that higher values always met this criterion. Scale scores were calculated as the average of each participant’s score within that category.

#### TV diary

To estimate the TV viewing of the preschoolers, parents were provided a seven-day TV diary. Parents were instructed to record whether their child was watching TV for 15-min periods from 6 a.m. to midnight each day. Among a non-Latino sample, parent-completed TV diaries of their children’s TV viewing had the highest correlation (*r* = 0.84) with the criterion standard of videotaped observation of child TV viewing compared to other methods of measuring TV viewing [29]. The TV diary also had good test-retest reliability (ICC = 0.82) among low-income Latino families and was correlated with the TV viewing measured by the TV allowance, an electronic meter measuring TV power (*r* = 0.45–0.55) and an Ecological Momentary Assessment (*r* = 0.47–0.51) [30].

#### TV Parenting Practices questionnaire

Parental mediation of children’s TV viewing was assessed using a 15-item questionnaire. Participants chose how frequently (never, rarely, sometimes, or often) they used a specific parenting practice, with each item corresponding to one of three mediation styles: Social Co-viewing, where parents and children watch TV together with no purpose but enjoyment (5 items); Instructional Mediation, where parents provide explanations or discuss elements of TV programs (5 items); and Restrictive Mediation, where parents set rules regarding acceptable program content and viewing duration (5 items) [30, 31]. The co-viewing subscale of the TV parenting practices survey was reverse coded, as co-viewing is positively correlated with child TV viewing [31]. The TV parenting practices combined score reflected a sum of the three subscale scores.

This scale was first developed and validated in a sample of Dutch parents of 5 to 12-year-old children [31]. It has since been validated for and used to assess the TV mediation practices of US parents of preschool and school-aged children, including predominantly Latino populations [32–34]. Cronbach’s alpha values were good for Social Co-viewing, Instructional Mediation, and Restrictive Mediation subscales and are listed respectively: Non-Latino populations (0.79, 0.80, 0.79) [31] and Latino populations (0.87, 0.81, 0.78) [34]. Additionally, previously reported regression analyses from our sample indicated that when accounting for child gender, age, parent BMI, child z-score, parent acculturation, and neighborhood disorder, Social Co-viewing was related to child TV viewing (*β* = 0.23) [35]. As the TV parenting practices score relates to child TV viewing while also capturing parental practices, it provides an important comparison for the validity of parental attitudes and beliefs measured in the MGDB.

### Statistical analyses

Baseline (Time 1) data for the total sample were used for all analyses, except test-retest reliability which used data at Time 1 and Time 2 and was limited to control group participants. Cronbach’s alpha measured internal consistency reliability for each MGDB scale, with an alpha level of 0.7 indicating acceptable reliability [36]. Exploratory Factor Analyses were conducted for scales with low internal consistency reliability. Pearson correlations and ICCs for absolute agreement using a two-way mixed-effects model [37–39] measured test-retest reliability on the control participant subsample comparing Time 1 and Time 2, separated by approximately two months. We assessed convergent validity through 1) Spearman correlations between MGDB scores at Time 1 and TV viewing minutes/day, and 2) Spearman correlations between the previously validated TV parenting practices scales [30–33] and the MGDB scales at Time 1. Spearman correlations were used due to the non-normal distribution of the data. Strengths of correlations were interpreted in line with previously published standards, with correlations below .3 considered negligible, .3 to .5 considered low correlations, .5 to .7 considered moderate correlations, and .7 and above considered high correlations [40]. For ICCs, values less than 0.5 indicated low reliability, 0.5 to 0.75 indicated moderate reliability, 0.75 to 0.9 indicated good reliability, and greater than 0.9 indicated excellent reliability [37]. Convergent validity was measured in two ways to assess whether the MGDB captures objective TV viewing of the child as well as parental behaviors. Construct validity was assessed via structural equation models. However, due to concerns that this study was exploratory and the first to evaluate the MGDB in the context of limiting children’s TV viewing, we evaluated construct validity of the MGDB scales divided into the Theory of Planned Behavior (attitudes, subjective norms, perceived behavioral control, and intentions) and non-Theory of Planned Behavior (positive and negative anticipated emotions, habits, self-efficacy, and desires) scales.

**Fig. 1:**
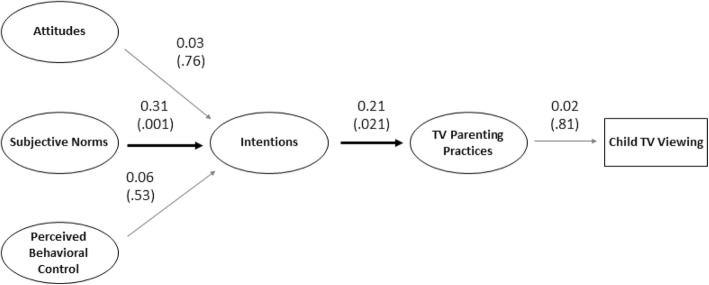
Diagram of model predicting child TV viewing from Theory of Planned Behavior MGDB scales. Not all paths are depicted for ease of presentation. Numbers outside parentheses indicate correlation values. Numbers inside parentheses indicate *p*-values

**Fig. 2:**
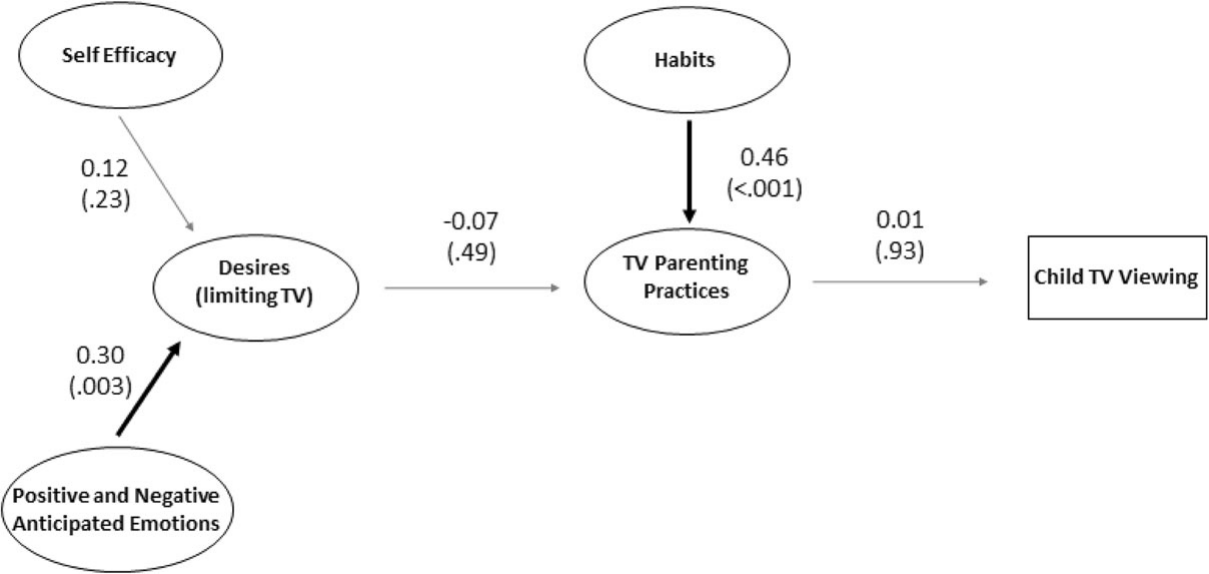
Diagram of model predicting child TV viewing from non-Theory of Planned Behavior MGDB scales. Not all paths are depicted for ease of presentation. Numbers outside parentheses indicate correlation values. Numbers inside parentheses indicate *p*-values

We conducted two structural equation models to assess construct validity and determine whether our MGDB items demonstrated a similar pattern of results to prior MGDB models. The first structural equation model evaluated the four MGDB scales derived from Theory of Planned Behavior (attitudes, subjective norms, perceived behavioral control, and intentions). Here, the motivational content of Attitudes, Perceived Behavioral Control, and Subjective Norms are converted by Intentions to influence TV parenting practices, which ultimately influence child TV viewing (Fig. 1). The second structural equation model evaluated the four non-Theory of Planned Behavior scales (self-efficacy, positive and negative anticipated emotions, desires, and habits). Here, Self-Efficacy and Positive and Negative Anticipated Emotions are converted by Desires to influence TV parenting practices, while Habits directly influence TV parenting practices, which ultimately impact child TV viewing (Fig. 2). Inclusion of all items in the models would be infeasible due to the large number of parameters. To reduce the number of parameters, we parceled each construct’s items into three groups. Parceling provides some advantages over item-level modeling, including parsimony*,* lower odds of correlated residuals, and reduced sampling error. Parcels were created using the item-to-construct balancing technique, which evenly distributes strong and weak items across parcels. Parcels then represent an aggregate-level indicator of the average of multiple items [41], which we used to conduct the structural equation models. We used Stata version 12 (Statacorp LP, College Station, TX) to conduct the analyses.

## Results

Characteristics of participants and their children can be found in Table 2. Mean age of children at Time 1 was 4.5 years for the intervention group (*n* = 93) and 4.3 years for the control group (*n* = 79). 46.2% of intervention children and 49.4% of control children were female.

**Table 2:** Table depicting demographic information of participants, separated into both control and intervention groups. Missing values indicate questions to which parents chose not to respond

	Intervention *n* = 93	Control *n* = 79
	Mean (SD)	Mean (SD)
Child age (years)	4.5 (0.4)	4.3 (0.6)
	N (%)	N (%)
Child sex		
Male	50 (53.8)	40 (50.6)
Female	43 (46.2)	39 (49.4)
	N (%)	N (%)
Child ethnicity		
Latino	93 (100)	79 (100)
Non-Latino	0 (0)	0 (0)
	N (%)	N (%)
Child race		
White	57 (61.3)	49 (62.0)
Other	28 (30.1)	19 (24.1)
Missing	8 (8.6)	11 (13.9)
	N (%)	N (%)
Child language		
Only Spanish	28 (30.1%)	25 (31.6%)
Spanish better than English	32 (34.4%)	32 (40.5%)
Both equally	16 (17.2%)	10 (12.7%)
English better than Spanish	17 (18.3%)	9 (11.4%)
Only English	0 (0.0%)	3 (3.8%)
	N (%)	N (%)
Parent’s sex		
Male	50 (53.8%)	39 (49.4%)
Female	43 (46.2%)	40 (50.6%)
	N (%)	N (%)
Parental education		
8th grade or less	41 (44.1)	34 (43.0)
Some high school	19 (20.4)	18 (22.8)
High school graduate or higher	33 (35.5)	24 (30.4)
Missing	0 (0)	3(3.8)
	N (%)	N (%)
Parent language		
Only Spanish	41 (44.1%)	38 (48.1%)
Spanish better than English	26 (28.0%)	19 (24.1%)
Both equally	19 (20.4%)	11 (13.9%)
English better than Spanish	5 (5.4%)	10 (12.7%)
Only English	1 (1.1%)	1 (1.3%)
Other	1 (1.1%)	0 (0.0%)

Internal consistency reliabilities for most MGDB scales were acceptable (Table 3). Most scales had a Cronbach’s alpha between 0.70 and 0.83, ranging from acceptable to good (Table 3), although two scales had Cronbach’s alpha values between 0.6 and 0.7 (Attitudes and Perceived Behavioral Control). The only exception was the 7-item Desires scale, which had a Cronbach’s alpha = 0.52. Due to the low internal consistency reliability, an Exploratory Factor Analysis was conducted for the Desires scale. This analysis indicated that a two-factor solution offered the best model fit and interpretability, with χ^2^(8) = 4.70; Root Mean Square Error of Approximation (RMSEA) = 0.00; 90% CI [0.00–0.92)]; Comparative Fit Index (CFI) = 1.00; Tucker-Lewis Index (TLI) = 1.00. Items 1–5 of the Desires scale loaded primarily on Factor 1 (Limiting TV), while items 6–7 loaded primarily on Factor 2 (Language Learning). These two factors had a low interscale correlation of − 0.063. Although the two items in Factor 2 were highly correlated (*r* = 0.72), we did not pursue a scale with so few items in further analyses. Thus, Desires Factor 1 was maintained for later analyses, and Factor 2 was excluded.

**Table 3:** Reliability and validity of the MGDB scales

MGDB scale	Cronbach’s alpha	Test-retest Pearson correlation among controls [95% CI]	Test-retest agreement: ICCs among controls [95% CI]	Spearman correlation with child TV viewing [95% CI]
Attitudes (n = 15)	0.62	0.44 [0.22–0.62]	0.45 [0.22–0.62]	− 0.11 [− 0.27–0.06]
Perceived behavioral control (n = 17)	0.61	0.28 [0.03–0.49]	0.28 [0.03–0.49]	− 0.10 [− 0.26–0.06]
Subjective norms (n = 9)	0.72	0.46 [0.24–0.64]	0.44 [0.22–0.62]	0.00 [− 0.16–0.17]
PNAE (*n* = 29)	0.77	0.44 [0.21–0.62]	0.42 [0.19–0.60]	− 0.02 [− 0.19–0.14]
Habits (*n* = 9)	0.79	0.59 [0.40–0.73]	0.59 [0.40–0.73]	− 0.14 [− 0.30–0.02]
Self-efficacy (n = 14)	0.83	0.35 [0.11–0.56]	0.35 [0.11–0.56]	− 0.08 [− 0.24–0.08]
Desires (n = 7)	0.52			
Desires Factor 1 (*n* = 5)	0.67	0.66 [0.48–0.78]	0.66 [0.49–0.78]	− 0.07 [− 0.23–0.10]
Intentions (*n* = 10)	0.70	0.45 [0.22–0.63]	0.45 [0.22–0.62]	− 0.06 [− 0.22–0.10]

Test-retest reliability using Pearson correlations was calculated for each of the MGDB scales. Results indicated that test-retest reliability was low to moderate and ranged from *r* = 0.35 to *r* = 0.66 (Table 3) with the exception of Perceived Behavioral Control which had a negligible test-retest correlation. Test-retest as measured by the ICC indicated moderate reliability for Habits and Desires, and poor reliability for the remaining scales.

Convergent validity was first assessed via Spearman correlation with TV viewing. Negligible correlation coefficient values across scales indicated low convergent validity. As a separate measure of convergent validity, Spearman correlations were conducted between the MGDB scales and the TV parenting practices scales. Multiple correlations were found between the TV parenting practices subscales and the MGDB scales of Habits and Self-Efficacy (Table 4). Of the three TV parenting practices mediation styles, Restrictive Mediation had the strongest correlation with the MGDB scales, *r* = 0.33 to *r* = 0.48. The TV parenting practices questionnaire total score was moderately correlated with the MGDB scale of Habits (Table 4).

**Table 4:** Spearman correlations [95% CI] between MGDB scores and TV parenting practices scores

MGDB Scale	TV parenting practices combined score	Co-viewing Sum	Instructional sum	Restrictive sum
Attitudes (*n* = 15)	0.04 [− 0.12–0.20]	− 0.07 [− 0.23–0.09]	0.02 [− 0.14–0.18]	0.11 [− 0.05–0.26]
Perceived behavioral control (*n* = 17)	0.29 [0.13–0.43]	− 0.04 [− 0.20–0.12]	0.19 [0.04–0.34]	0.27 [0.11–0.41]
Subjective norms (n = 9)	0.08 [− 0.08–0.24]	− 0.12 [− 0.27–0.05]	0.14 [− 0.02–0.29]	0.10 [− 0.06–0.26]
PNAE (n = 29)	0.00 [− 0.16–0.16]	− 0.15 [− 0.31–0.01]	0.11 [− 0.05–0.26]	0.06 [− 0.10–0.22]
Habits (n = 9)	0.51 [0.37–0.62]	− 0.10 [− 0.25–0.06]	0.31 [0.16–0.45]	0.48 [0.35–0.59]
Self-efficacy (n = 14)	0.26 [0.11–0.41]	−0.25 [− 0.39- -0.09]	0.24 [0.08–0.38]	0.33 [0.18–0.46]
Desires Factor 1 (n = 5)	0.04 [−0.12–0.20]	0.07 [− 0.09–0.22]	− 0.04 [− 0.20–0.12]	0.00 [− 0.16–0.16]
Intentions (*n* = 10)	0.17 [0.01–0.32]	−0.16 [− 0.32–0.00]	0.17 [0.01–0.32]	0.22 [0.06–0.36]

Two structural equation models were used to determine whether the factor structure of the MGDB items fit our predicted model, and therefore was similar to previous MGDBs (e.g., [21]). The first structural equation model was used to assess the fit of the model including the four Theory of Planned Behavior MDGB scales (attitudes, subjective norms, perceived behavioral control, and intentions; Fig. 1). Additionally, indicators for the school of the participants’ child were included as covariates with all constructs to account for the cluster-randomized nature of this study (students were clustered within 6 schools). Overall model fit was acceptable (*x*^2^ (97) = 113.65; *p* = .119; RMSEA = .03; CFI = 0.98; TLI = 0.97). However, considering the low Cronbach’s alpha for two of the scales in this model (attitudes and perceived behavioral control), we ran a likelihood ratio test comparing the structural equation model with and without the low-reliability scales. The sensitivity analysis revealed that the full model (including all scales) provided better model fit (*x*^2^ (11)=204.16; *p* < .001).

The second structural equation model assessed the fit of a model including the four non-Theory of Planned Behavior MGDB scales (self-efficacy, positive and negative anticipated emotions, desires factor 1, and habits; Fig. 2). This analysis was also conducted using parcels and including school of the participants’ child as covariates. The structural equation model revealed that overall model fit was questionable ((*x*^2^(97) = 125.39; *p* = .028; RMSEA = 0.04; CFI = 0.97; TLI = 0.96), yielding an acceptable RMSEA, CFI, and TLI, but non-acceptable model *x*^2^. Considering the low Cronbach’s alpha for desires factor 1, we ran a likelihood ratio test comparing the structural equation model with and without this scale. The sensitivity analysis revealed that the full model (including all scales) provided better model fit (*x*^2^ (6)=80.46; *p* < .001).

**Fig. 3:**
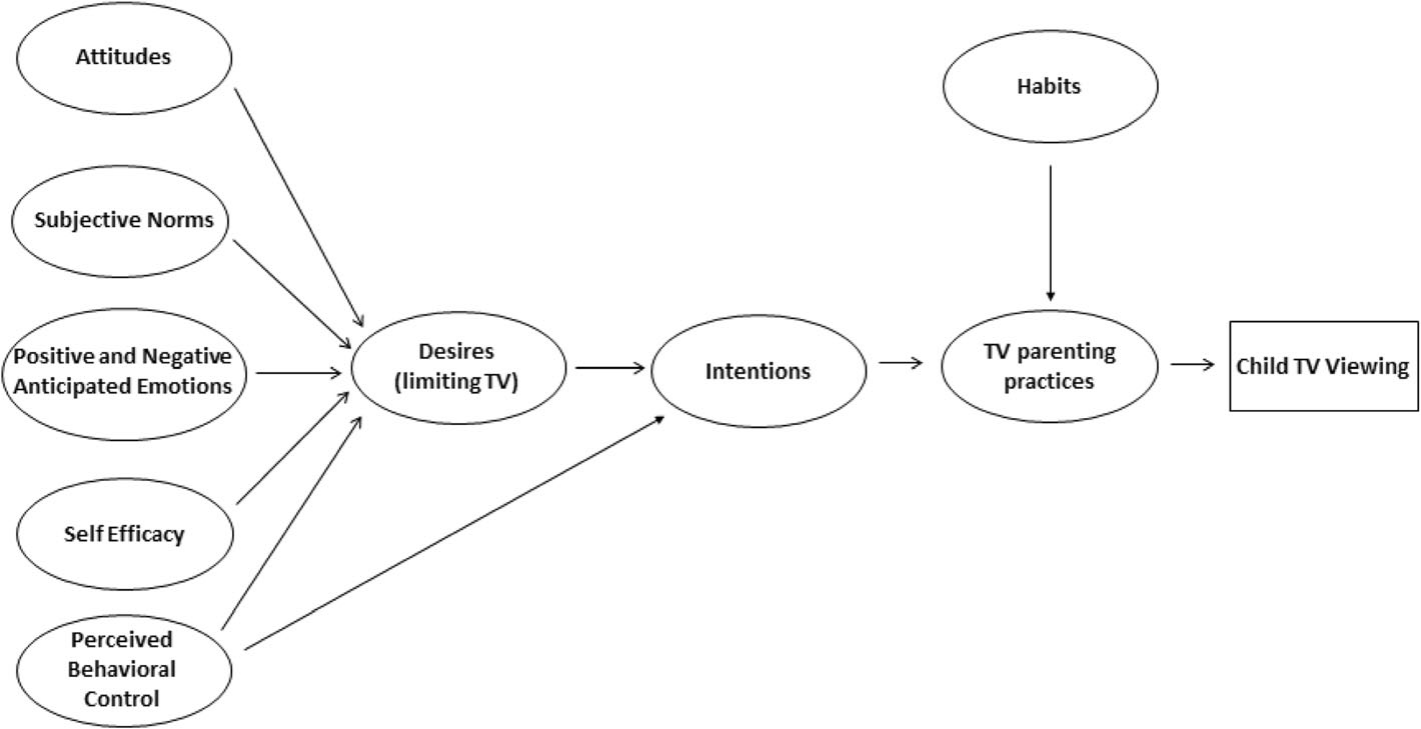
Diagram of full model relating MGDB scales, TV parenting practices, and child TV viewing. This model was not analyzed, but represents conceptually how these constructs may fit together

Figure 3 depicts a theoretical model of how the Theory of Planned Behavior and non-Theory of Planned Behavior scales may fit together with TV parenting practices to predict child TV viewing, although this model was not directly analyzed.

## Discussion

This is the first set of scales to measure parental attitudes and beliefs toward controlling their child’s TV viewing. The MGDB scales characterizing Latino parents’ attitudes and beliefs for limiting their preschoolers’ TV viewing generally had acceptable internal consistency reliability (although Attitudes, Perceived Behavioral Control, and Desires Factor 1 were below a Cronbach’s alpha of 0.7). The pattern of acceptable internal consistency reliability for most, but not all, of the MGDB scales appears similar to initial psychometric assessment of the previous Model of Goal Directed Vegetable Parenting Practices [21], indicating that the present MGDB may be a useful measure, but additional refinement of items within specific scales is likely needed to improve reliability. This is also important given that the two scales which demonstrated convergent validity with TV Parenting Practices were not high in reliability. Habits showed moderate test-retest correlations and moderate ICC, while Self-Efficacy showed low test-retest correlations and poor ICC. Thus, refinement of items to improve reliability for these scales may be beneficial.

Additionally, results revealed medium test-retest reliability and convergent validity compared to TV parenting practices for two out of the eight scales. However, none of the scales demonstrated convergent validity with child TV viewing. We speculate that this may be because a more complex model (e.g., Fig. 3) is necessary in order to understand how all of these scales may work in concert to predict TV parenting practices, which ultimately predict children’s TV viewing (rather than each scale individually directly predicting child TV viewing). This question remains to be addressed by a study with a sample large enough to have full confidence in the results.

The Desires scale had the lowest internal consistency reliability, but was revealed to have two distinct factors. Only two of the MGDB scales were correlated with TV parenting practices subscales, and Restrictive Mediation had the strongest correlation to the MGDB scales. This seems to indicate that Restrictive Mediation is an important aspect of the MGDB scale for low-income Latino populations, and therefore the MDGB may be particularly informative for this TV parenting practice. Future research should investigate whether more of the MGDB scales correlate with TV parenting practices and child TV viewing in a sample large enough to have full confidence in the results.

The first structural equation model from the Theory of Planned Behavior variables was found to have acceptable model fit, indicating that these MGDB scales may function similarly to previously reported Theory of Planned Behavior scales (e.g., [23]). However, the second structural equation model from the non-Theory of Planned Behavior variables had questionable model fit, with acceptable RMSEA, CFI, and TLI, but non-acceptable model *x*^2^. This pattern of results is in contrast to previous MGDB research which has demonstrated improved model fit for non-TPB over TPB items for measuring attitudes and beliefs toward physical activity [25]. However, the *x*^2^ test is more susceptible to Type 1 errors with small sample sizes. This may account for the discrepancy across the 4 fit indices for the non-Theory of Planned Behavior model, but future studies with larger sample sizes will be necessary to clarify this issue. Thus, the results of the non-Theory of Planned Behavior model may be informative for future research, but with the present sample size should be interpreted with caution. Although we split our structural equation models into two separate analyses due to our small sample size while Perugini & Bagozzi [23] used one structural equation model, the results provide similar insight into how best to measure parental attitudes and beliefs to impact child health behavior. Thus, our results suggest that this model may be useful for better understanding parental attitudes and beliefs toward limiting their child’s TV viewing, and that a survey intended for parents of Latino preschool-aged children, specifically, may be beneficial.

Limitations to this study include low generalizability due to recruiting from only one city, parceling to reduce the number of parameters being estimated, and self-report by parents which is necessary to address their attitudes and intentions, but may introduce the possibility of a social desirability response bias. Furthermore, data collection occurred prior to the AAP’s adjusted recommendation that children aged 2–5 watch no more than 1 h of TV per day [17], which may limit how these findings generalize to a more restrictive set of guidelines. Additionally, the number of exploratory analyses conducted may have inflated our Type I error. Further, the 2-month period between test and retest is longer than many other psychometric studies, and therefore may have biased these results toward the null hypothesis. Future studies are necessary to confirm and extend validity and reliability for each MGDB scale with a larger and broader sample, and longitudinal research is necessary to establish causality. Longitudinal research may be helpful for investigating the relation among MGDB scales, TV parenting practices, and child TV viewing, as perhaps additional time is necessary to allow for an observable change in the child’s behavior. The MGDB questionnaire in this study included 105 items, which allowed for a thorough investigation into each of the 8 scales. However, future research may identify whether the length of the MGDB could be shortened for easier use in interventions or community settings. To do so, item response modeling should be used to cut redundant items without impacting internal consistency reliability. Future research can also investigate how these findings extend to other forms of sedentary screen and media use.

## Conclusion

The MGDB scales may offer valuable information for assessing Latino parents’ attitudes and beliefs for limiting their preschoolers’ TV viewing, a particularly important behavior to target as TV viewing is still the dominant type of screen time among young children [42]. Overall, our results suggest that the MGDB scales show potential, but are in need of further refinement and investigation. In the future, the MGDB may be useful for assessing why Latino children engage in more TV viewing than their peers [12]. Aside from MGDB’s use for assessment of behavioral constructs, these scales could inform the development of interventions and policies targeting Latino parents’ attitudes and beliefs for limiting their preschool children’s TV viewing. It may be especially useful for informing modification of TV parenting practices, which are influential to children’s TV viewing behaviors [43]. Such modifications are much-needed for this population which is particularly at risk for excessive TV viewing [10] and childhood obesity [2].

## Supplementary information

**Additional file 1.** Model of Goal Directed Behavior questionnaire.

## Data Availability

Datasets used in the current study will be made available upon request.

## Abbreviations

AAP: American Academy of Pediatrics
CFI: Comparative Fit Index
MGDB: Model of Goal Directed Behavior
PNAE: Positive and Negative Anticipated Emotions
RMSEA: Root Mean Square Error of Approximation
TLI: Tucker-Lewis Index
TV: television
US: United States
