# Supplementary material for: Model of goal directed behavior for limiting Latino preschoolers’ television viewing: validity and reliability
Source: BMC Public Health. 2020 Feb 5;20:185. doi: 10.1186/s12889-020-8268-x (PMC7003399; doi:10.1186/s12889-020-8268-x)
Supplement: Supplementary file 1 — Additional file 1. Model of Goal Directed Behavior questionnaire. [file 12889_2020_8268_MOESM1_ESM.docx]

Additional file 1

**Instructions:**

- **You will be given a set of questions.**
- **Please select the answer you think is the best in regard to your 2-5 year old child.**
- **Your answers are confidential; there are no rights or wrong answers to any of the questions.**

**A. ATTITUDES**

*Please read each statement carefully. Select the answers that you think is the best in relation with your* ***2-5 year old child.*** *There are no right or wrong answers.*

*How much do you agree or disagree with the following statements?*

| **If your child watched less TV on most days, your child would:** | | **Agree**  **(1)** | **Disagree**  **(2)** | **Neither Agree or Disagree**  **(3)** |
| --- | --- | --- | --- | --- |
|  | … be healthier. | □ | □ | □ |
|  | … have less difficulty paying attention. | □ | □ | □ |
|  | … be exposed to fewer violent or aggressive TV programs. | □ | □ | □ |
|  | … have more energy to play. | □ | □ | □ |
|  | … have fewer problems becoming an overweight child. | □ | □ | □ |
|  | … be exposed to fewer adult issues on TV, like sexual situations. | □ | □ | □ |
|  | … get more physical activity. | □ | □ | □ |
|  | … think better. | □ | □ | □ |
|  | … learn better eating habits. | □ | □ | □ |
|  | … set a good example for others. | □ | □ | □ |
|  | … be too thin. | □ | □ | □ |
|  | …make you spend less money on junk food or fast food. | □ | □ | □ |
|  | …be exposed to fewer unhealthy TV programs or commercials. | □ | □ | □ |
|  | …be better behaved. | □ | □ | □ |
|  | …live longer. | □ | □ | □ |

**B. PERCEIVED POSITIVE/NEGATIVE BEHAVIORAL CONTROL**

*Please read each statement carefully. Select the answers that you think is the best in relation with your* ***2-5 year old child.*** *There are no right or wrong answers.*

*How easy or difficult is for you the following statements?*

| **How easy would it be to get your child to watch less TV …** | | **Easy (1)** | **Difficult (2)** | **Neither easy nor difficult (3)** |
| --- | --- | --- | --- | --- |
|  | …by removing the TV from their bedroom or playroom. | □ | □ | □ |
|  | …by turning off the TV more often. | □ | □ | □ |
|  | …by reading books to him/her instead. | □ | □ | □ |
|  | …by telling your child to turn off the TV when he/she is watching an unsuitable program. | □ | □ | □ |
|  | …by setting specific viewing hours for your child. | □ | □ | □ |
|  | …by forbidding your child to watch certain programs. | □ | □ | □ |
|  | ...by restricting the amount of time children watch TV. | □ | □ | □ |
|  | ...by specifying in advance the programs that may be watched. | □ | □ | □ |
|  | …by playing games with them instead. | □ | □ | □ |
|  | …by letting them play outside instead. | □ | □ | □ |
|  | …by watching less TV yourself. | □ | □ | □ |
|  | …without promising your child a reward. | □ | □ | □ |
|  | …without removing the TV from his/her bedroom or playroom. | □ | □ | □ |
|  | …without physically struggling with your child. | □ | □ | □ |
|  | …without begging your child to watch less TV. | □ | □ | □ |
|  | …without yelling at your child. | □ | □ | □ |
|  | …without watching less TV yourself. | □ | □ | □ |

**C. SUBJECTIVE NORMS**

***Mark with a “1”, “2” and “3” the three people who most influence your decisions about your child in a good or bad way:***

|  |  | Spouse or partner |  |  | Grandmother |
| --- | --- | --- | --- | --- | --- |
|  |  | Mother |  |  | Grandfather |
|  |  | Mother in law |  |  | Sister/brother |
|  |  | Father |  |  | Sister in law/brother in law |
|  |  | Father in law |  |  | Close friends |
|  |  | Caregiver/Babysitter/Nanny |  |  |  |

*Please read each statement carefully. Select the answers that you think is the best in relation with your* ***2-5 year old child.*** *There are no right or wrong answers.*

*How much do you agree or disagree with the following statements?*

| **Do you agree or disagree with the following statements:** | | **Agree**  **(1)** | **Disagree (2)** | **Neither Agree nor Disagree**  **(3)** |
| --- | --- | --- | --- | --- |
|  | **Referring to the person marked with a “1” above.** It is important to **this person** that my child watches 2 hours or less of TV most days. | □ | □ | □ |
|  | **Referring to the person marked with a “1” above.** It is important to me to please **this person** when it comes to getting my child to watch 2 hours or less of TV most days. | □ | □ | □ |
|  | **Referring to the person marked with a “2” above.** It is important to **this person** that my child watches 2 hours or less of TV most days. | □ | □ | □ |
|  | **Referring to the person marked with a “2” above.** It is important to me to please **this person** when it comes to getting my child to watch 2 hours or less of TV each day. | □ | □ | □ |
|  | **Referring to the person marked with a “3” above.**. It is important to **this person** that my child watches 2 hours or less of TV most days. | □ | □ | □ |
|  | **Referring to the person marked with a “3” above.**. It is important to me to please **this person** when it comes to getting my child to watch 2 hours or less of TV each day. | □ | □ | □ |
|  | Most parents are trying to get their child to watch less TV most days. | □ | □ | □ |
|  | Most children are watching 2 hours or less of TV most days. | □ | □ | □ |
|  | Most parents have their child watching 2 hours or less of TV most days. | □ | □ | □ |

**D. POSITIVE AND NEGATIVE ANTICIPATED EMOTIONS**

*Please read each statement carefully. Select the answers that you think is the best in relation with your* ***2-5 year old child.*** *There are no right or wrong answers.*

*How much do you agree or disagree with the following statements?*

| **A. If you asked your child to watch less TV and they did it, you would feel:** | | **Agree**  **(1)** | **Disagree (2)** | **Neither Agree nor Disagree**  **(3)** |
| --- | --- | --- | --- | --- |
|  | …pleased | □ | □ | □ |
|  | ...disappointed | □ | □ | □ |
|  | …happy | □ | □ | □ |
|  | ...proud | □ | □ | □ |
|  | …frustrated | □ | □ | □ |
|  | …excited | □ | □ | □ |
|  | …concerned | □ | □ | □ |
|  | …upset | □ | □ | □ |

| **B. If you asked your child to watch less TV and they refused, you would feel:** | | **Agree (1)** | **Disagree (2)** | **Neither Agree nor Disagree (3)** |
| --- | --- | --- | --- | --- |
|  | …pleased | □ | □ | □ |
|  | ...disappointed | □ | □ | □ |
|  | …happy | □ | □ | □ |
|  | ...proud | □ | □ | □ |
|  | …frustrated | □ | □ | □ |
|  | …excited | □ | □ | □ |
|  | …concerned | □ | □ | □ |
|  | …upset | □ | □ | □ |

| **C. If you allowed your child to watch a TV program that he/she likes, and he/she refuses to watch it, you would feel:** | | **Agree**  **(1)** | **Disagree**  **(2)** | **Neither Agree nor Disagree (3)** |
| --- | --- | --- | --- | --- |
|  | …surprised | □ | □ | □ |
|  | ...proud | □ | □ | □ |
|  | ...happy | □ | □ | □ |
|  | ...pleased | □ | □ | □ |
|  | …excited | □ | □ | □ |
|  | ...confused | □ | □ | □ |

*Please read each statement carefully. Select the answers that you think is the best in relation with your* ***2-5 year old child.*** *There are no right or wrong answers.*

*How much do you agree or disagree with the following statements?*

| **D. If you allowed your child to watch a TV program he/she dislikes, and he/she watches it, you would feel:** | | **Agree a lot**  **(1)** | **Agree a little**  **(2)** | **Disagree a little**  **(3)** |
| --- | --- | --- | --- | --- |
|  | …disappointed | □ | □ | □ |
|  | …frustrated | □ | □ | □ |
|  | …upset | □ | □ | □ |
|  | ...confused | □ | □ | □ |
|  | …challenged | □ | □ | □ |
|  | …concerned | □ | □ | □ |
|  | …not surprised | □ | □ | □ |

**E. HABITS**

*Please read each statement carefully. Select the answers that you think is the best in relation with your* ***2-5 year old child.*** *There are no right or wrong answers.*

*How often you do the following things?*

| **How often do you do this without thinking about it?** | | **Always**  **(1)** | **Sometimes**  **(2)** | **Never**  **(3)** |
| --- | --- | --- | --- | --- |
|  | Turn off the TV. | □ | □ | □ |
|  | Read books to your child instead of watching TV. | □ | □ | □ |
|  | Tell your child to turn off the TV when he/she is watching an unsuitable program. | □ | □ | □ |
|  | Set specific viewing hours for your child. | □ | □ | □ |
|  | Forbid your child to watch certain programs. | □ | □ | □ |
|  | Restrict the amount of TV your child can watch. | □ | □ | □ |
|  | Specify in advance the programs that may be watched. | □ | □ | □ |
|  | Negotiate with your child the programs that he/she can watched | □ | □ | □ |
|  | Negotiate with your child the hours when he/she can watch TV | □ | □ | □ |

**F. SELF-EFFICACY**

*Please read each statement carefully. Select the answers that you think is the best in relation with your* ***2-5 year old child.*** *There are no right or wrong answers.*

| **How sure are you that you can…** | | **Sure**  **(1)** | **Somewhat sure**  **(2)** | **Not sure**  **(3)** |
| --- | --- | --- | --- | --- |
|  | …limit your child’s TV watching to 2 hours or less daily. | □ | □ | □ |
|  | …overcome problems in getting your child to watch TV for 2 hours or less daily. | □ | □ | □ |
|  | …offer different activities for your child instead of TV. | □ | □ | □ |
|  | …limit your child’s access to TV, such as removing a TV from your child’s bedroom or playroom. | □ | □ | □ |
|  | … get your child to eat dinner without watching TV. | □ | □ | □ |
|  | …play games with your child instead of watching TV. | □ | □ | □ |
|  | … read books with your child instead of watching TV. | □ | □ | □ |
|  | … watch TV with your child. | □ | □ | □ |
|  | … get your child to play outside instead of watching TV. | □ | □ | □ |
|  | …prepare meals with your child instead of watching TV. | □ | □ | □ |
|  | …teach your child English instead of watching TV. | □ | □ | □ |
|  | …teach your child Spanish instead of watching TV. | □ | □ | □ |
|  | …choose TV programs that your child can watch. | □ | □ | □ |
|  | …set specific hours when your child can watch TV. | □ | □ | □ |

**G. DESIRES**

*Please read each statement carefully. Select the answers that you think is the best in relation with your* ***2-5 year old child.*** *There are no right or wrong answers.*

| **Do you agree or disagree with the following statements:** | | **Agree**  **(1)** | **Disagree**  **(2)** | **Neither Agree nor Disagree**  **(3)** |
| --- | --- | --- | --- | --- |
|  | I want my child to watch less TV | □ | □ | □ |
|  | I want my child to eat more meals with the TV off | □ | □ | □ |
|  | I want to remove the TV from my child's bedroom or playroom | □ | □ | □ |
|  | I want to specify in advance the programs that may be watched | □ | □ | □ |
|  | I want to restrict the amount of TV my child can watch | □ | □ | □ |
|  | I want my child to watch TV to learn more English | □ | □ | □ |
|  | I want my child to watch TV to learn more Spanish | □ | □ | □ |

**H. INTENTIONS**

*Please read each statement carefully. Select the answers that you think is the best in relation with your* ***2-5 year old child.*** *There are no right or wrong answers.*

*How definitive are you that you are able to do the following things?*

| **In the next month you are planning to…** | | **Will do**  **(1)** | **May**  **or**  **May not do**  **(2)** | **Will not do**  **(3)** |
| --- | --- | --- | --- | --- |
|  | ... tell your child to turn off the TV when he/she is watching an unsuitable program. | □ | □ | □ |
|  | ... set specific viewing hours for your child. | □ | □ | □ |
|  | ... forbid your child to watch certain programs. | □ | □ | □ |
|  | …restrict the amount of TV your child can watch. | □ | □ | □ |
|  | …specify in advance the programs that may be watched**.** | □ | □ | □ |
|  | … play games with your child instead. | □ | □ | □ |
|  | … let your child play outside instead. | □ | □ | □ |
|  | … watch less TV yourself. | □ | □ | □ |
|  | … negotiate with your child the programs that he/she can watched | □ | □ | □ |
|  | … negotiate with your child the hours when he/she can watch TV | □ | □ | □ |
